# *Borneocola* (Zingiberaceae), a new genus from Borneo

**DOI:** 10.3897/phytokeys.75.9837

**Published:** 2016-11-29

**Authors:** Yen Yen Sam, Atsuko Takano, Halijah Ibrahim, Eliška Záveská, Fazimah Aziz

**Affiliations:** 1 Forest Research Institute Malaysia, 52109 Kepong, Selangor, Malaysia; 2 Museum of Nature and Human Activities, Hyogo 6 chome, Yayoigaoka, Sanda, Hyogo 669-1546, Japan; 3 Institute of Biological Sciences, Faculty of Science, University of Malaya, 50603 Kuala Lumpur, Malaysia; 4 Institute of Botany, University of Innsbruck, Austria; 5 Department of Aquatic Science, Universiti Malaysia Sarawak, 94300 Kota Samarahan, Sarawak, Malaysia; 6 Yen Yen Sam

**Keywords:** Distichochlamys, Myxochlamys, Scaphochlamys, morphology, phylogeny, taxonomy

## Abstract

A new genus from Borneo, *Borneocola* Y.Y.Sam, is described here. The genus currently contains eight species previously classified as members of the *Scaphochlamys* Baker. The finding is based on the results of the morphological and molecular studies of *Scaphochlamys* throughout its geographical range and its closely allied sister groups, *Distichochlamys* M.F.Newman and *Myxochlamys* A.Takano & Nagam. *Borneocola* is nested within the tribe Zingibereae and its monophyly is strongly supported by both ITS and matK sequence data. The genus is characterised by several thin, translucent and marcescent floral bracts, absence of coloured streaks on the labellum and capitate stigma with two dorsal knobs. The genus is distributed in northwest Borneo and all species are very rare and highly endemic.

## Introduction

Southeast Asia is the centre of diversity for the family Zingiberaceae. Here, new taxa are continuously being discovered and named, both at the generic and specific levels. Several of the recent discoveries were further supported by the phylogenetic analyses which give a better understanding of the evolutionary relationships within the family (Kress and Larsen 2001; Kress et al. 2010; Leong-Škorničková et al. 2011). During the revision of the genus *Scaphochlamys* throughout its entire geographical range by the first author, some distinctive morphological traits were observed in several Bornean species, suggesting they might represent a separate group from the Peninsular Malaysian taxa. This hypothesis was confirmed by the phylogenetic analyses which are presented here and the eight species previously included in the genus *Scaphochlamys* are recircumscribed in this paper as a new genus, *Borneocola* Y.Y.Sam.

The genus *Scaphochlamys* was described by Baker (1892) in the Flora of British India with *Scaphochlamys malaccana* Baker from Mt. Ophir (now known as Gunung Ledang), Peninsular Malaysia, chosen as the type species. Holttum (1950) carried out the first comprehensive revision of the genus in which he recognised 19 species, all of which were recorded in the peninsula. When Smith (1987) reviewed the tribe Hedychieae in Borneo, she applied the generic delimitation defined by Holttum and recognised five *Scaphochlamys* species in Borneo. Out of the five, *Scaphochlamys polyphylla* and *Scaphochlamys petiolata* were formerly placed in the genus *Haplochorema* K.Schum. Sakai and Nagamasu (2006) discovered that *Haplochorema gracilipes* K.Schum. also have the characteristics of *Scaphochlamys* and effected the transfer. Recent years have seen a surge in the new species discovered from Borneo bringing the total number of Bornean *Scaphochlamys* to 14 (Poulsen and Searle 2005, Meekiong et al. 2011, Ooi and Wong 2014; Meekiong 2015).

*Distichochlamys* M.F.Newman and *Myxochlamys* A.Takano & Nagam. are sister genera to *Scaphochlamys* with several unique characteristics clearly separating them from *Scaphochlamys* (Newman 1995, Searle and Hedderson 2000, Kress et al. 2002, Ngamriabsakul et al. 2004, Takano and Nagamasu 2007). However, the distinction, based on morphological characters, became ambiguous as several taxa described recently exhibit exceptions to the usual generic characters. For example, *Scaphochlamys calcicola* A.D.Poulsen & R.J.Searle, a species named in 2005 from Sarawak, has a distichous inflorescence, a distinguishing character for the genus *Distichochlamys* M.F.Newman. Larsen and Newman (2001) also reported another *Scaphochlamys* species with a distichous inflorescence from north Peninsular Malaysia. A current study on the morphology of *Scaphochlamys* also revealed that some species display the characteristics of *Distichochlamys* and *Myxochlamys*. To test the validity of the current generic concept of *Scaphochlamys* and closely related genera *Distichochlamys* and *Myxochlamys*, we have examined their relationship by utilising ITS and matK markers together with the analysis of the morphology across these genera.

## Materials and methods

### Morphological study

The morphological study was based on living plants in the forest, cultivated plants in the nursery of the Forest Research Institute Malaysia and specimens in the herbaria of AAU, BKF, C, FI, E, K, KEP, KLU, PSU, SAN, SAR and SING. A total of 372 herbarium specimens were examined in this study which includes 29 *Scaphochlamys* species and four *Borneocola* species (the types of another four *Borneocola* species were not yet deposited in the herbaria).The morphological characters examined in the study were habit; position of the rhizome, thickness and colour; height of leafy stem, its base (whether swollen to form a bulbous base); distance between leafy stems; characters of bladeless sheath such as colour, indumentum, number and length; ligule length, indumentum and shape; petiole length, indumentum, whether channelled or rounded in cross section; number of leaves per leafy stem; lamina colour on both surfaces, size, shape, venation, texture, indumentum, apex and base; length of the inflorescence and infructescence, arrangement of the floral bracts on the rachis, characters of floral bracts and bracteoles (colour, indumentum, texture, shape); size, colour and shape of calyx, floral tube, corolla lobes, labellum, staminodes, stamen, ovary.

### DNA extraction, amplification and sequencing

Fresh leaves from the cultivated plants or silica-dried materials from plants collected in the field were used for genomic DNA extraction.

For the ITS, the genomic DNA was extracted using the DNeasy Plant Mini Kit (Qiagen, Valencia, California, USA) following the manufacturer’s protocol. Two primers, ITS 5P (5’-GGAAGGAGAAGTCGTAACAAGG-3’) and ITS 8P (5’-CACGCTTCTCCAGACTACA3’) (Moller and Cronk 1997) were used to amplify the ITS region during the polymerase chain reaction (PCR). The thermal cycle of PCR for the amplification of the ITS sequences is initial denaturation at 94°C for 2 minutes, 40 cycles of denaturation at 94°C for 30 seconds, primers annealing at 48°C for 2 minutes, an extension at 72°C for 45 seconds and final extension at 72°C for 7 minutes. The PCR products were then purified using MinElute Gel Extraction Kit (Qiagen, Valencia, California, USA).

For the matK, the protocols for DNA extraction, condition, purification and DNA sequencing were described previously by Takano and Nagamasu (2007). The PCR and sequencing primers for matK (cpDNA) were TA-240f (5’-GGGAAA GGATGGGGTCTCCCG-3’), TA-150r (5’-CTCAAGGAGTTTTGTGGTTC-3’), TA-470F (5’-CCCTCTCCCGTCCATATGGA-3’) (all three were designed in the present study), matK8 (Steele and Vilgalys 1994), m5r (Kress et al. 2002), matK8r (Ooi et al. 1995), trnK2621 (Liston and Kadereit 1995), TA-10F, TA-05R, TA-02F and TA-02R (all from Takano and Okada 2002).

### Sequence alignment and phylogenetic analysis

Raw sequence data were assembled and edited manually using BioEdit software ver. 7.2.5 (Hall 1999). DNA sequences were aligned with the CLUSTALW 1.83 software package, with default settings and multiple alignments (Thompson et al. 1994). Alignments of the matK sequences of cpDNA and the ITS sequences of nrDNA were combined. Gaps were deleted.

A total of 100 individuals including 54 taxa of *Scaphochlamys* and allied species were used. The three *Siphonochilus* species were used as an outgroup (Kress et al. 2002). Materials, accession numbers for the sequences, vouchers and references to the literature are presented in Table 1 at the end of this paper. Three datasets which comprise ITS, matK and ITS+matK combined, each containing 82, 78, and 61 taxa, were constructed. These three datasets were analysed using three methods: maximum parsimony, maximum likelihood and Bayesian analysis. A maximum parsimony (MP) analysis was performed with MEGA 6 (Tamura et al. 2013). Heuristic searches were conducted with RANDOM addition, SPR branch swapping and MULPARS options. Support for each branch was estimated with a bootstrap analysis, with 1000 replications (Felsenstein 1985), in a heuristic search with RANDOM addition and TBR branch swapping. The maximum likelihood (ML), based on the Tamura-Nei model (Tamura and Nei 1993), was also determined with MEGA 6 (Tamura et al. 2013). Neighbor-Join and BioNJ algorithms were applied to a matrix of pairwise distances estimated with the maximum composite likelihood approach; then, the topology that had the best log likelihood value was selected. Bootstrap analysis under the MP criterion was conducted with “fast” stepwise, addition searches, with 1000 replicates. In addition, a Bayesian analysis was carried out with MrBayes software ver. 3.1.2 (Huelsenbeck and Rohnquist 2001; Rohnquist and Huelsenbeck 2003). The best fitting substitution model (the GTR+G model for nrDNA datasets, the GTR+G model for cpDNA datasets and the GTR+I+G model for cpDNA+nrDNA datasets) was selected for Bayesian analysis based on a series of hierarchical likelihood ratio tests, implemented in MrModeltest software ver. 2.3 (Nylander 2004). The analysis was performed with the selected model and two simultaneous runs of two million generations with four chains, sampling every 100 generations. Each analysis reached stationarity (i.e. when the average standard deviation of split frequencies between runs was ≤ 0.01) well before the end of the run. Burn-in trees were discarded and the remaining trees and their parameters were saved. A 50% majority rule consensus tree was constructed. The results of the Bayesian analysis were reported as the posterior probability (PP; Huelsenbeck and Rohnquist 2001), which is equal to the percentage of phylogenetic trees sampled when a given clade was resolved. Only PP scores above 50% are shown.

**Table 1.** List of accession details, vouchers and references used in the phylogenetic analyses.

No	Subfamily	Tribe	Species	ITS	matK	References/Voucher
1	*Alpinioideae* Link	*Alpinieae* A.Rich.	*Alpinia blepharocalyx* K.Schum.	AF478709	AF478809	Kress et al. 2002
2			*Alpinia elegans* K.Schum.	AF478713	AF478813	Kress et al. 2002
3			*Amomum villosum* Lour.	–	AF478824	Harris et al. 2000 (ITS), Kress et al. 2002 (matK)
4			*Amomum yunnanense* S.Q.Tong	AY352012	–	Xia et al. 2004
5			*Elettariopsis kerbyi* R.M.Sm.	AF414496	AF478845	Pedersen 2004 (ITS)/Kress et al. 2002 (matK)
6			*Renealmia battenbergiana* Cummins ex Baker	AF478779	AF478880	Kress et al. 2002
7	*Siphonochileae* W.J.Kress	*Siphonochileae* W.J.Kress	*Siphonochilus aethiopicus* (Schweinf.) B.L.Burtt	AF478792	AF478893	Kress et al. 2002
8			*Siphonochilus decorus* (Druten) Lock	AF478793	AF478894	Kress et al. 2002
9			*Siphonochilus kirkii* (Hook.) B.L.Burtt	AF478794	AF478895	Kress et al. 2002
10	*Tamijioideae* W.J.Kress	*Tamijieae* W.J.Kress	*Tamijia flagellaris* S.Sakai & Nagam.	AF478797	AF478898	Kress et al. 2002
11	*Zingiberoideae* Haask.	*Globbeae* Meisn.	*Gagnepainia thoreliana* K.Schum.	AF478752	AF478851	Kress et al. 2002
12			*Hemiorchis rhodorrhachis* K.Schum.	AY339706	AY341090	Williams et al. 2004
13			*Mantisia wengeri* C.E.C.Fischer	–	AF478871	Kress et al. 2002
14	*Zingiberoideae* Haask.	*Zingibereae* Meisn.	*Boesenbergia pulcherrima* Kuntze	AF478725	AF478825	Kress et al. 2002
15			*Boesenbergia rotunda* (L.) Mansf.	AF478727	AF478826	Kress et al. 2002
16			*Borneocola biru* (Meekiong) Y.Y.Sam	–	LC148403	FRI 50290 (KEP)
17			*Borneocola calcicola* (A.D.Poulsen & R.J.Searle) Y.Y. Sam	LC148062	LC148380	FRI 50290 (KEP)
18			*Borneocola* sp. FRI 50295	LC148085	LC148404	FRI 50295 (KEP)
19			*Borneocola* sp. S 99106	LC148086	LC148405	S 99106 (SAR)
20			*Borneocola stenophyllus* (Ooi & S.Y.Wong) Y.Y.Sam	LC148084	LC148400	FRI 50288 (KEP)
21			*Borneocola petiolatus* (K.Schum.) Y.Y.Sam	LC148075	LC148395	FRI 50291 (KEP)
22			*Borneocola reticosus* (Ridl.) Y.Y.Sam	LC148078	LC148398	FRI 50294 (KEP)
23			*Camptandra parvula* Ridl.	AF478730	AF478830	Kress et al. 2002
24			*Caulokaempferia saxicola* K.Larsen	AY478732	AF478831	Kress et al. 2002
25			*Cautleya gracilis* (Sm.) Dandy	AF478734	AF478833	Kress et al. 2002
26			*Cautleya spicata* Baker	AF478735	AF478834	Kress et al. 2002
27			*Cornukaempferia aurantiflora* J.Mood & K.Larsen	AF478736	AF478835	Kress et al. 2002
28			*Curcuma bicolor* J.Mood & K.Larsen	AF478737	AF478837	Kress et al. 2002
29			*Curcuma roscoeana* Wall.	AF478739	AB047741	Kress et al. 2002 (ITS)/Cao et al. unpublished (matK)
30			*Distichochlamys citrea* M. F. Newman	AY424757	–	Ngamriabsakul et al. 2004
31			*Distichochlamys citrea* M. F. Newman 2	AB552946	AB552951	Ngamriabsakul 24 (E)
32	*Zingiberoideae* Haask.	*Zingibereae* Meisn.	*Distichochlamys* sp. AS18	AB552947	AB553309	Adele Smith 18 (E)
33			*Distichochlamys* sp. Kress01-6848	AF478745	AF478844	Kress et al. 2002
34			*Haniffia albiflora* K.Larsen & J.Mood	AF478756	AF478855	Kress et al. 2002
35			*Hedychium longicornutum* Griff. ex Baker	AF478761	AF478860	Kress et al. 2002
36			*Hedychium villosum* Wall.	AF478762	AF478861	Kress et al. 2002
37			*Hitchenia glauca* Wall.	AF478765	AF478864	Kress et al. 2002
38			*Kaempferia parviflora* Wall.	–	AB232052	Searle and Hedderson 2000
39			*Kaempferia rotunda* L.	AF478767	AF478868	Kress et al. 2002
40			*Kaempferia* sp. Kress98-6289	AF478768	AF478869	Kress et al. 2002
41			*Myxochlamys mullerensis* A.Takano & Nagam.	AB245522	AB269791	Takano and Nagamasu 2007
42			*Myxochlamys nobilis* Nagam. ined.	AB552948	AB553310	Nagamasu 8274 (BO, KYO)
43			*Pommereschea lackneri* Wittm.	–	AF478877	Kress et al. 2002
44			*Pyrgophyllum yunnanense* (Gagnep.) T.L.Wu & Z.Y.Chen	AF478777	AF478878	Kress et al. 2002
45			*Rhynchanthus beesianus* W.W.Sm.	AF478784	AF478885	Kress et al. 2002
46			*Roscoea cautleoides* Gagnep.	AF478736	AF478887	Kress et al. 2002
47			*Roscoea purpurea* Sm.	AF478787	AF478888	Kress et al. 2002
48			*Scaphochlamys abdullahii* Y.Y.Sam & Saw	LC148054	–	FRI 44375 (KEP)
49			*Scaphochlamys abdullahii* Y.Y.Sam & Saw	LC148055	LC148374	FRI 50198 (KEP)
50			*Scaphochlamys atroviridis* Holttum	LC148056	–	FRI 68924 (KEP)
51			*Scaphochlamys baukensis* Y.Y.Sam	LC148057	–	FRI 68955 (KEP)
52			*Scaphochlamys biloba* (Ridl.) Holttum	LC148059	–	FRI 46606 (KEP)
53			*Scaphochlamys biloba* (Ridl.) Holttum	LC148081	–	FRI 50224 (KEP)
54			*Scaphochlamys biloba* (Ridl.) Holttum	LC148083	–	FRI 66331 (KEP)
55			*Scaphochlamys biloba* (Ridl.) Holttum 1	AF478788	AY478889	Kress et al. 2002
56			*Scaphochlamys biloba* (Ridl.) Holttum 2	AF202416	–	Wood et al. 2000
57			*Scaphochlamys breviscapa* Holttum	–	LC148377	FRI 50269 (KEP)
58			*Scaphochlamys breviscapa* Holttum	LC148060	LC148376	FRI 44984 (KEP)
59			*Scaphochlamys burkillii* Holttum	–	LC148379	FRI 68928 (KEP)
60			*Scaphochlamys burkillii* Holttum	LC148061	–	FRI 46504 (KEP)
61			*Scaphochlamys concinna* (Baker) Holttum	AJ388283	–	Searle and Hedderson 2000
62			*Scaphochlamys concinna* (Baker) Holttum	LC148063	LC148381	FRI 50351 (KEP)
63			*Scaphochlamys cordata* Y.Y.Sam & Saw	LC148064	–	FRI 44306 (KEP)
64			*Scaphochlamys endauensis* Y.Y.Sam & Ibrahim	–	LC148383	FRI 50243 (KEP)
65			*Scaphochlamys endauensis* Y.Y.Sam & Ibrahim	LC148080	–	FRI 50218 (KEP)
66			*Scaphochlamys erecta* Holttum	LC148065	–	FRI 44987 (KEP)
67			*Scaphochlamys grandis* Holttum	–	LC148384	FRI47184 (KEP)
68			*Scaphochlamys grandis* Holttum	LC148066	LC148385	FRI 50171 (KEP)
69	*Zingiberoideae* Haask.	*Zingibereae* Meisn.	*Scaphochlamys johorensis* Y.Y.Sam	LC148082	–	FRI 66566 (KEP)
70			*Scaphochlamys klossii* (Ridl.) Holttum	LC148067	LC148387	FRI 50238 (KEP)
71			*Scaphochlamys kunstleri* (Baker) Holttum	AF478789	AY478890	Kress et al. 2002
72			Scaphochlamys kunstleri (Baker) Holttum var. rubra C.K.Lim	AB552950	AB553312	Anon C 8003 & C. Ngamriabsakul 25 (E)
73			Scaphochlamys kunstleri (Baker) Holttum var. kunstleri	–	LC148388	FRI 68926 (KEP)
74			Scaphochlamys kunstleri (Baker) Holttum var. kunstleri	LC148068	–	FRI 68936 (KEP)
75			Scaphochlamys kunstleri var. speciosa C.K.Lim	–	LC148389	FRI 68936 (KEP)
76			*Scaphochlamys lanceolata* (Ridl.) Holttum	LC148069	LC148390	FRI 50130 (KEP)
77			*Scaphochlamys laxa* Y.Y.Sam & Saw	–	LC148391	FRI 68961 (KEP)
78			*Scaphochlamys longifolia* (Ridl.) Holttum	LC148070	LC148392	FRI 47065 (KEP)
79			*Scaphochlamys malaccana* Baker	–	LC148393	FRI 50203 (KEP)
80			*Scaphochlamys malaccana* Baker	LC148071	–	FRI 50208 (KEP)
81			*Scaphochlamys minutiflora* Jenjitt.& K.Larsen	–	LC148394	3175
82			*Scaphochlamys obcordata* P.Sirirugsa & K.Larsen	AJ388286	–	Searle and Hedderson 2000
83			*Scaphochlamys oculata* (Ridl.) Holttum	LC148072	LC148396	FRI 50262 (KEP)
84			*Scaphochlamys pennipicta* Holttum	LC148073	–	FRI 50261 (KEP)
85			*Scaphochlamys perakensis* Holttum	LC148074	–	FRI 50214 (KEP)
86			*Scaphochlamys polyphylla* (K.Schum.) B.L.Burtt & R.M.Sm.	LC148076	LC148397	FRI 50289 (KEP)
87			*Scaphochlamys pusilla* Y.Y.Sam	LC148077	–	FRI 50260 (KEP)
88			*Scaphochlamys rubromaculata* Holttum	–	LC148399	FRI 50178 (KEP)
89			*Scaphochlamys rubromaculata* Holttum	LC148079	LC148378	FRI 50172 (KEP)
90			*Scaphochlamys samunsamensis* Meekiong & Hidir	–	LC148401	MK 2344 (HUMS)
91			*Scaphochlamys* sp.nov.	–	LC148402	FRI 68983 (KEP)
92			*Scaphochlamys sub-biloba* (Burkill ex Ridl.) Holttum	–	LC148375	FRI 75334 (KEP)
93			*Scaphochlamys sylvestris* (Ridl.)Holttum	LC148087	–	FRI 50197 (KEP)
94			*Scaphochlamys tenuis* Holttum	LC148088	–	FRI 47233 (KEP)
95			*Schaphochlamys cf. gracilipes* (K.Schum.) S.Sakai & Nagam.	–	LC148386	K.Meekiong (HUMS)
96			*Smithatris supraneanae* W.J.Kress & K.Larsen	AF478795	AF478896	Kress et al. 2002
97			*Stahlianthus involucratus* (King ex Baker) R.M.Sm.	AF478796	AF478897	Kress et al. 2002
98			*Zingiber gramineum* Noronha	AF478800	AF478902	Kress et al. 2002
99			*Zingiber sulphureum* Burkill ex I.Theilade	AF478801	AF478904	Kress et al. 2002
100			*Zingiber wrayii* Prain ex Ridl.	AF478802	AF478905	Kress et al. 2002

## Results

### Phylogenetic analyses

**Figure 1.**
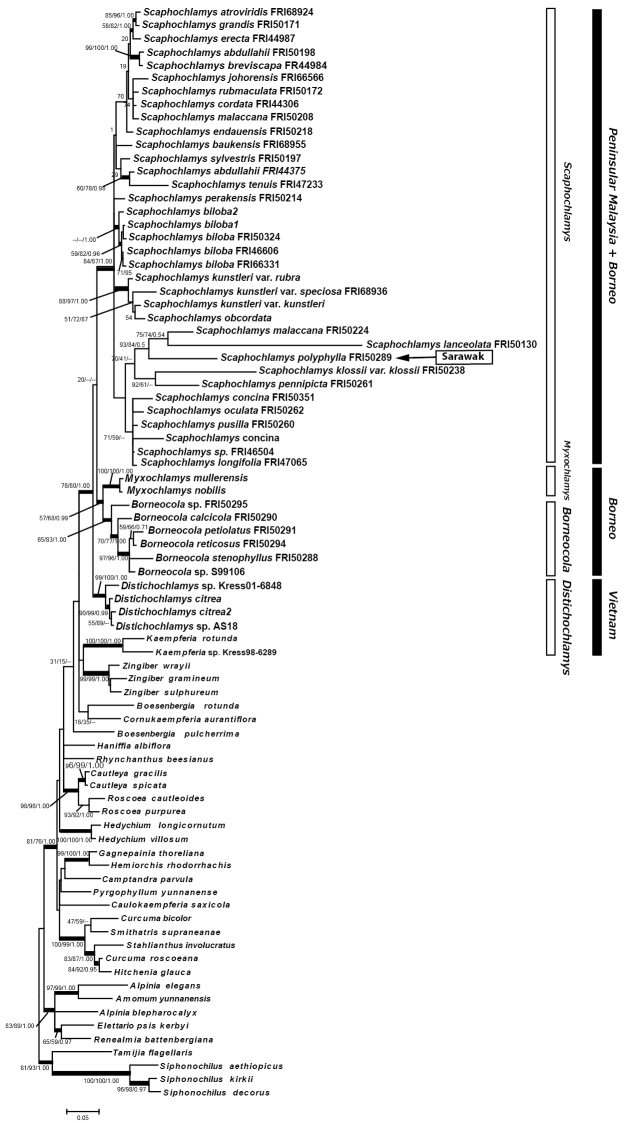
Molecular phylogenetic analysis of the ITS sequence data by the Maximum Likelihood method. Numbers above branches indicate bootstrap values of ML and MP and posterior probability of Bayesian Analysis.

The ITS datasets for 82 individuals with 29 taxa of *Scaphochlamys* and 6 taxa of *Borneocola* contained 786 characters after alignment, which decreased to 769 after gaps were deleted; 319 of these were parsimony-informative. Likelihood analysis resulted in a ML tree with –lnL = 10438.212. Parsimony analysis produced three parsimonious trees with 1865 steps, a consistency index (CI) of 0.391 and retention index (RI) of 0.609. The ML, MP and Bayesian trees had similar topology; the ML tree is shown with bootstrap (BS) and MP-BS, and Bayesian Posterior Probability (PP) support in Figure 1 below.

*Scaphochlamys* formed a well supported clade (ML-BS/MP-BS/BA-PP support, 84/87/1.00). Each *Myxochlamys* and *Borneocola* consisted of a well supported subclade and became sisters to each other and they also became sistersto the *Scaphochlamys* clade. *Distichochlamys* species formed a well supported subclade and became sister to the *Myxochlamys* + *Scaphochlamys* + *Borneocola* clade (ML-BS/MP-BS/BA-PP 99/100/1.00).

**Figure 2.**
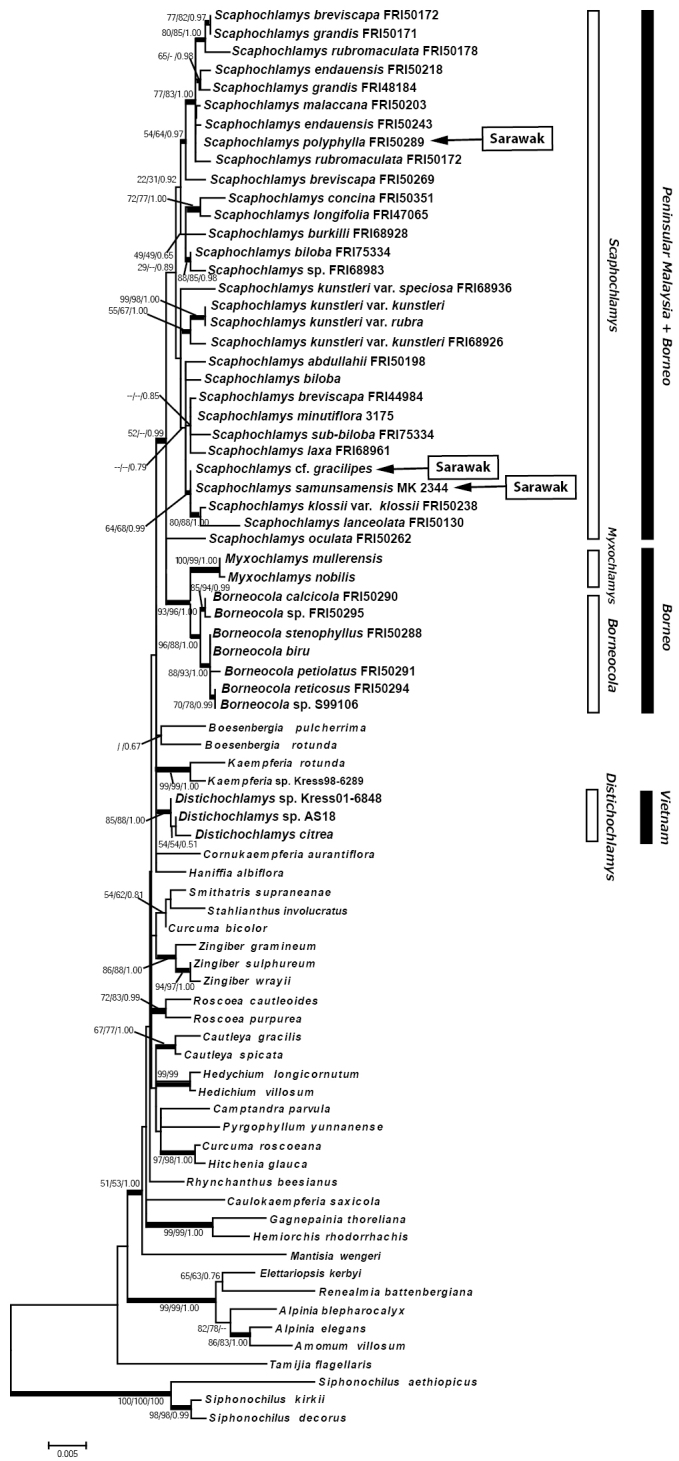
Molecular phylogenetic analysis of the matK sequence data by the Maximum Likelihood method. Numbers above branches indicate bootstrap values of ML and MP and posterior probability of Bayesian Analysis.

The matK datasets for 78 individuals including 25 taxa of *Scaphochlamys* and 7 taxa of *Borneocola* contained 1,599 characters after alignment; 182 of these were parsimony-informative. Likelihood analysis resulted in a ML tree with -lnL = 5952.438. Parsimony analysis produced ten parsimonious trees with 557 steps, a consistency index (CI) of 0.613 and retention index (RI) of 0.080. The ML, MP and Bayesian trees had similar topology; the ML tree is shown with BS and MP-BS, PP support in Figure 2 below.

Each of the two *Myxochlamys* species and seven *Borneocola* species formed a strongly supported subclade and became sisters to each other. *Scaphochlamys* became sister to them, but bootstrap or probability support was weak. The *Distichochlamys* species formed a well supported subclade, but all the genera that belong to subfamily Zingiberoideae became sisters to *Scaphochlamys* + *Myxochlamys* + *Borneocola* clade and not only to *Distichochlamys*.

**Figure 3.**
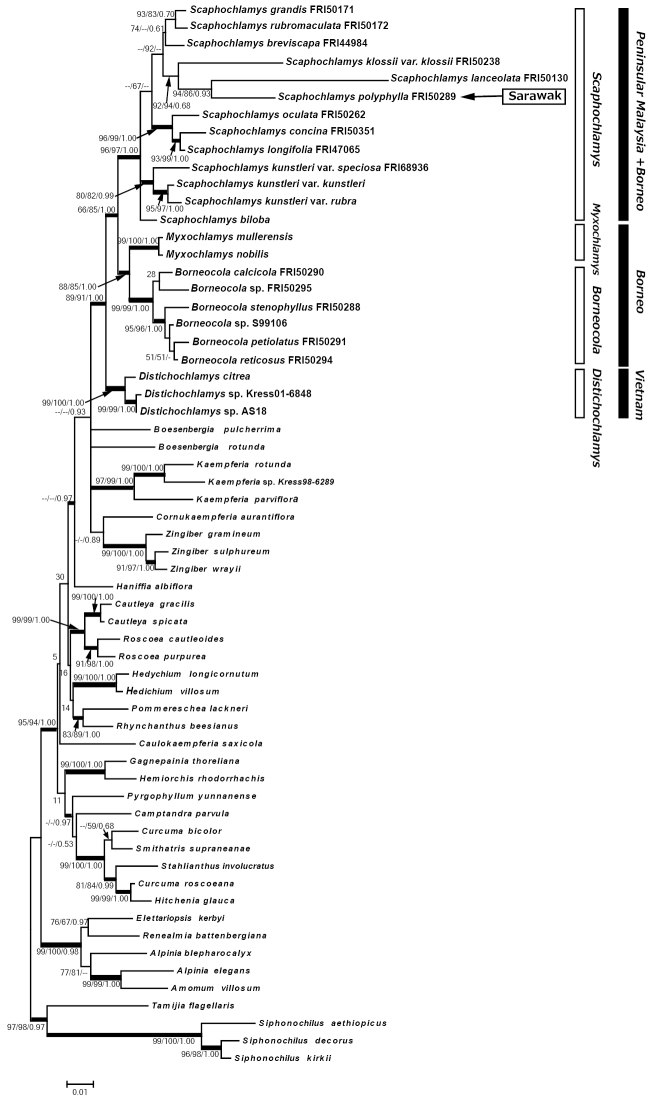
Molecular phylogenetic analysis of the ITS+matK sequence data by the Maximum Likelihood method. Numbers above branches indicate bootstrap values of ML and MP and posterior probability of Bayesian Analysis.

The combined ITS and matK datasets for 61 individuals including 13 taxa of *Scaphochlamys* and 6 taxa of *Borneocola*, resulted in 2,336 characters, 488 of these were parsimony-informative (Figure 3 below). Likelihood analysis resulted in a ML tree with lnL = 16671.531. Parsimony analysis produced the most parsimonious trees with 2247 steps, a CI of 0.440 and a RI of 0.635. The ML, MP strict consensus and Bayesian trees had almost the same topology; the ML tree is shown with MP-BS, ML-BS and BA/PP support in Figure 3.

Two *Myxochlamys* species and six *Borneocola* species formed a strongly supported subclade each and became sisters to each other. *Scaphochlamys* became sister to them and the bootstrap or posterior probability support was moderate. *Distichochlamys* species formed a well supported subclade and became sister to *Scaphochlamys* + *Myxochlamys* + *Borneocola* clade.

### Morphology

**Figure 4.**
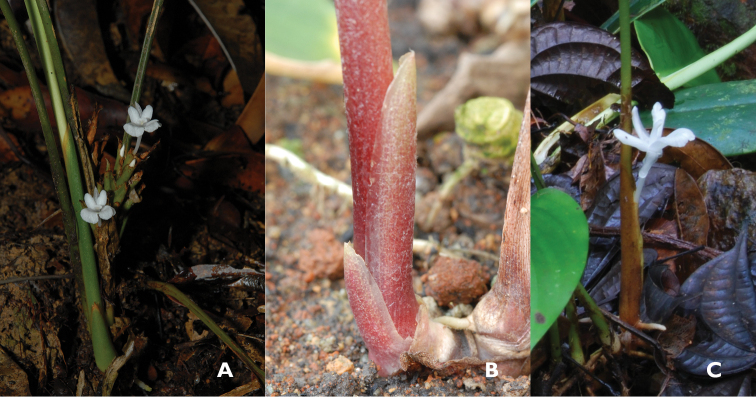
Bladeless sheaths **A** Green and coriaceous in *Scaphochlamys klossii* (Peninsular Malaysia) **B** Red and coriaceous in *Scaphochlamys abdullahii* (Peninsular Malaysia) **C** Papery and marcescent in *Borneocola calcicola* (Sarawak). (Photographs by Y.Y. Sam).

The *Borneocola* and *Scaphochlamys* species look similar in their vegetative morphologies. They are mostly small-sized gingers without the conspicuous pseudostem, with one to several leaves arranged spirally and tightly on a very short stem at the base. So far, all the *Borneocola* species examined are unifoliate. Similarly, most of the *Scaphochlamys* species also bear one leaf except for several species which have leafy shoots composed of multiple leaves, for example, *Scaphochlamys grandis*, *Scaphochlamys lanceolata*, *Scaphochlamys kunstleri*, *Scaphochlamys malaccana* and *Scaphochlamys minutiflora*. The basal part of the leaves is covered with a few bladeless sheaths which are rather different for both groups in terms of their texture and colour. For *Scaphochlamys*, the sheaths are coriaceous, green, green with a red tinge or red and mostly persistent until the end of flowering (Figure 4A, B). On the other hand, the sheaths of *Borneocola* are thinner in texture with a lighter shade of green or brown. The thin sheaths normally dry up early (Figure 4C) and sometimes they are completely shredded during the time of flowering.

**Figure 5.**
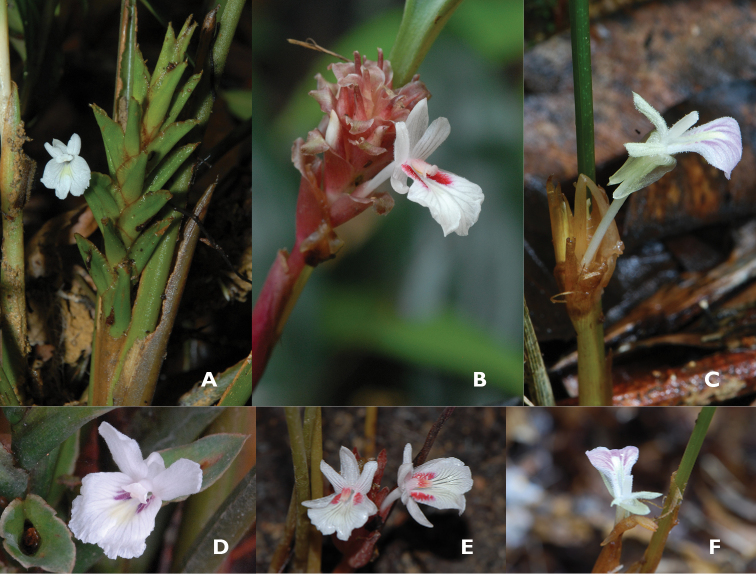
**A–C** Floral bracts **A** Green and coriaceous in *Scaphochlamys klossii* **B** Red and coriaceous in *Scaphochlamys pusilla* **C** Scarious and marcescent in *Borneocola petiolatus* **D–F** Variegation on labellum **D** White labellum with purple lines beside the median band in *Scaphochlamys malaccana* **E** White labellum with red streaks beside the band in *Scaphochlamys concinna* **F** Lilac labellum without coloured streaks beside the band in *Borneocola petiolatus*. (Photographs by Y.Y. Sam)

The inflorescences of *Borneocola* and *Scaphochlamys* are terminal, stalked and consisted of few to many floral bracts. The differences lie in the characteristics of the floral bracts and flowers. *Borneocola* species have thin, translucent, early decaying and marcescent floral bracts. The colours of the bracts can be pink, pale brown, pale or light green (Figure 5A). On the contrary, the bracts of *Scaphochlamys* are coriaceous and sometimes hard in texture. They are usually green, green tinged red, red or reddish brown and remain fresh throughout the flowering (Figure 5B, C).

Besides the characteristics of the floral bracts, the variegation on the labellum can give a quick guide to the two genera. Most *Scaphochlamys* have white flowers with a yellow median band and lilac, purple, red streaks or patches flanking the band on the labellum (Figure 5D, E). However, there is no such variegation on the labellum of *Borneocola* (Figure 5F). The whole labellum of *Borneocola* is pale pink, lilac, violet or white with a light yellow or greenish yellow median band.

**Figure 6.**
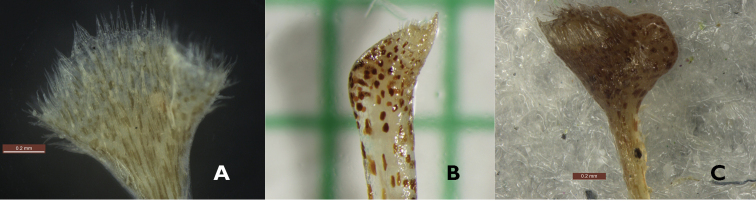
Stigma **A** Funnel-shaped in *Scaphochlamys endauensis* **B** Beak-like in *Scaphochlamys biloba* **C** Capitate in *Borneocola petiolatus*. (Photographs by **A** & **C** N.M. Aidil, **B** Y.Y. Sam)

Both *Borneocola* and *Scaphochlamys* have a long slender floral tube which is mostly puberulent externally in *Borneocola* (except for *Borneocola calcicola*) but glabrous for *Scaphochlamys*. Another marked difference observed is in the stigma shape. *Scaphochlamys* has a funnel-shaped or beak-like stigma (Figure 6A, B) while it is almost oblate with two dorsal knobs in *Borneocola* (Figure 6C).

## Taxonomic treatment

### Borneocola

Taxon classificationPlantaeZingiberalesZingiberaceae

Y.Y.Sam gen. nov.

urn:lsid:ipni.org:names:77158811-1

#### Diagnosis.

Similar to *Scaphochlamys* and *Myxochlamys*. *Borneocola* has thin, translucent and marcescent floral bracts, absence of coloured streaks on labellum and two dorsal knobs on the stigma versus the coriaceous and persistent floral bracts, coloured streaks on labellum and absence of dorsal knobs on the stigma in *Scaphochlamys*. The mucilage on the floral bracts and the versatile anther of *Myxochlamys* are absent in *Borneocola*.

#### Type species.

*Borneocola reticosus* (Ridl.) Y.Y.Sam, comb. nov. *Gastrochilus reticosa* Ridl., J. Straits Branch Roy. Asiat. Soc. 44: 195 (1905).

#### Description.

Terrestrial rhizomatous herb, evergreen, rarely exceeding 50 cm in height. Rhizome creeping on the ground, terminal decumbent, rhizome elements short or long; roots fine, extensive, not tuberous. Leafy stem unifoliate, enclosed by a few bladeless sheaths at base, bladeless sheaths linear, papery, glabrous to hairy, light green or light brown, decaying early, leaf sheath glabrous or hairy, base swollen, margin thin and narrow; ligule membranous, inconspicuous, decaying early; petiole channelled in cross section, glabrous, lamina narrowly ovate to elliptic, rarely oblong, asymmetric, margin entire, smooth.

Inflorescence flowering from base to apex; peduncle short, usually hidden within leaf sheath; spike composed of compact rachis and 2–5 (–13) fertile bracts, bracts spirally and closely overlapping (rarely distichous), boat-shaped, 2-keeled, pink, pale brown, pale or light green, thin, translucent, glabrous or hairy, decaying early, marcescent, amplexicaul at the base of the bract, cincinni compact, 2–3 flowers in each cincinnus. First bracteole directly opposite floral bract and enclosing all the flowers and subsequent bracteoles, linear-shaped, 2-keeled, shorter than bracts, rarely same length. Flowers thin, delicate, ephemeral. Calyx tubular, splitting unilaterally on one side, floral tube long slender, usually puberulent externally, inner surface with a groove enveloping the style, corolla lobes 3, triangular ovate, translucent, glabrous, dorsal lobe apex hooded, lateral lobes 2, narrower than dorsal lobe. Staminodes elliptic to narrowly obovate, white, light yellow or green, spreading laterally, lined with translucent veins from base to apex, covered with glandular hairs on adaxial surface. Labellum obovate, flat, bilobed distally, rarely entire, translucent veins spread from base to apical part, pale white, pink, lilac or violet, median band light yellow or greenish yellow, without coloured streaks or patches beside the band, adaxial surface covered with glandular hairs. Stamen bends forward over labellum, usually white and covered with glandular hairs on abaxial surface, filament grooved, short, anther theacae 2, linear, dehiscing along entire length, basal ending with or without short spur, anther crest short, not recurved. Ovary ellipsoid, glabrous or sparsely hairy, unilocular, with basal placentation, less than 10 locules. Stigma small, held at apex of thecae, near oblate, 2 dorsal knobs, ostiole forward facing, ciliate. Epigynous glands 2, filiform, yellow. Fruit a capsule, ellipsoid or ovoid. Seeds not seen.

#### Etymology.

This new genus is named after the island of Borneo and -*cola* (Latin) means dweller or inhabitant. This is to recognise the extremely rich and unique biodiversity that is found in Borneo.

#### Distribution.

Borneo. The genus is currently known to occur only in the northwest and possibly central Borneo. Eight species are recorded from Sarawak, Malaysia and many more are undescribed.

#### Key to *Borneocola* species (modified from Ooi and Wong 2014)

**Table:** 

1	Adaxial lamina with distinctly raised tessellate venation	**6 *Borneocola reticosus***
–	Adaxial lamina without distinctly raised tessellate venation	**2**
2	Lamina broadly oblanceolate and elliptic to oblong, lateral veins conspicuously raised on adaxial surface	**1 *Borneocola argenteus***
–	Lamina linear, lanceolate to broadly ovate and elliptic, lateral veins not conspicuously raised on adaxial surface	**3**
3	Lamina linear to very narrowly lanceolate, < 3 cm wide	**8 *Borneocola stenophyllus***
–	Lamina lanceolate, ovate to elliptic, > 3 cm wide	**4**
4	Leaf sheath plus petiole < 10 cm long; lamina apex broadly acute to rounded, base cuneate	**4 *Borneocola iporii***
–	Leaf sheath plus petiole > 10 cm long; lamina apex acute to acuminate, base attenuate, cordate, rounded or truncated	**5**
5	Lamina lanceolate to ovate, < 7 cm wide	**6**
–	Lamina elliptic to broadly elliptic and ovate, > 7 cm wide	**7**
6	Basal lamina rounded to truncated; inflorescence stalk 3–9 cm long; labellum pale lilac	**5 *Borneocola petiolatus***
–	Basal lamina attenuate; inflorescence stalk 1–2 cm long; labellum purplish blue	**2 *Borneocola biru***
7	Inflorescence about 3 cm long; labellum pale pink	**7 *Borneocola salahuddinianus***
–	Inflorescence > 7 cm long; labellum white	**3 *Borneocola calcicola***

### 1. Borneocola argenteus

Taxon classificationPlantaeZingiberalesZingiberaceae

(R.M.Sm.) Y.Y.Sam comb. nov.

urn:lsid:ipni.org:names:77158824-1

Scaphochlamys argentea R.M.Sm., Notes Roy. Bot. Gard. Edinburgh 44: 209 (1987).
Scaphochlamys depressa Mas Izzaty, A.Ampeng & K.Meekiong, Folia Malaysiana 14(2): 19 (2013).

#### Type.

MALAYSIA. Sarawak, First Division, Lundu, near foot of Gunung Perigi, 6 Aug 1962, Burtt B2700 (holotype: E!).

#### Notes.

It is one of the most distinctive species, either in the field or herbarium sheet. This dainty plant has a long creeping rhizome and well spaced leafy shoots, prominently stiff lateral veins raised on its adaxial lamina, both on living plants and dried specimens.

*Scaphochlamys depressa* Mas Izzaty, Ampeng & Meekiong is unmistakably the same as *Scaphochlamys argentea* with its prominent raised lateral veins, broadly elliptic lamina and well spaced leafy shoots. Meekiong (2015) explained that the inflorescence of *Scaphochlamys depressa* which exerted from the petiole is different from *Scaphochlamys argentea* where the inflorescence emerges from the base of the petiole. This observation is incorrect as all gingers have terminal inflorescences.

### 2. Borneocola biru

Taxon classificationPlantaeZingiberalesZingiberaceae

(Meekiong) Y.Y.Sam comb. nov.

urn:lsid:ipni.org:names:77158812-1

Scaphochlamys biru Meekiong, Folia Malaysiana 16(1): 37 (2015).

#### Type.

MALAYSIA. Sarawak, Kuching Division, Matang Wildlife Centre, 21 May 2014 Meekiong et al. s.n. (holotype: SAR; isotype: Herbarium, Universiti Malaysia Sarawak. Types not yet deposited as of 5 May 2016).

#### Notes.

*Borneocola biru* is the most recent species described from Sarawak. It has a deep purplish blue labellum, different from all other *Borneocola* species which are white or in lighter shades.

### 3. Borneocola calcicola

Taxon classificationPlantaeZingiberalesZingiberaceae

(A.D.Poulsen & R.J.Searle) Y.Y.Sam comb. nov.

urn:lsid:ipni.org:names:77158813-1

Scaphochlamys calcicola A.D.Poulsen & R.J.Searle, Gard. Bull. Singapore 57: 29 (2005).

#### Type.

MALAYSIA. Sarawak, Kuching Division, Bau area, Gunung Tai Ton, 1°24'N, 110°8'E, 20 June 2003, Poulsen, Jugah & Clausager 2022 (holotype: SAR!; isotypes: AAU, E!, K!, L).

#### Notes.

*Borneocola calcicola* is the largest amongst the *Borneocola* species. Poulsen and Searle (2005) observed that the distichous inflorescence is one of the characteristics of the plant. However, a recent collection of *Borneocola calcicola*, Sam FRI 50290, from Seromah, Bau, showed spirally arranged floral bracts. There was a mixture of spirally and distichously arranged floral bracts in its population in Bau, Sarawak.

### 4. Borneocola iporii

Taxon classificationPlantaeZingiberalesZingiberaceae

(Meekiong & A.Ampeng) Y.Y.Sam comb. nov.

urn:lsid:ipni.org:names:77158814-1

Scaphochlamys iporii Meekiong & A.Ampeng, Folia Malaysiana 12(1): 19 (2011).

#### Type.

MALAYSIA. Sarawak, Kapit, Lanjak Entimau Wildlife Sanctuary, Bukit Menyarin, 3 April 2008, Meekiong MK1839 (holotype: SAR; isotype Herbarium, Universiti Malaysia Sarawak. Types not yet deposited as of 5 May 2016).

#### Notes.

*Borneocola iporii* is a small ginger creeping on the humus rich forest floor. It is most similar to *Borneocola argenteus* with both having a unifoliate shoot, leafy shoots far apart, broad lamina, short inflorescence and compact rachis. However, the conspicuously raised lateral veins of *Borneocola argenteus* can readily distinguish it from *Borneocola iporii*.

### 5. Borneocola petiolatus

Taxon classificationPlantaeZingiberalesZingiberaceae

(K.Schum.) Y.Y.Sam comb. nov.

urn:lsid:ipni.org:names:77158815-1

Haplochorema petiolatum K.Schum. in Engler, Pflanzenr. IV, 46 (Heft 20): 90 (1904). Scaphochlamys petiolata (K.Schum.) R.M.Sm., Notes Roy. Bot. Gard. Edinburgh 44: 210 (1987).

#### Type.

MALAYSIA. Sarawak, First Division, Mt. Singhi (= Gunung Singai), Dec 1892, Haviland 2026 (lectotype: K! designated by Searle 2010; isolectotype: E!, SAR!).

#### Notes.

*Borneocola petiolatus* is distinguished by its long petiole and narrow leaves from the other species. Its lamina length is almost 3 times the width (12–21.5 × 3.1–7.1 cm). Smith (1987) found that *Borneocola petiolatus* has small inflorescences as in *Borneocola argenteus*. However, both can be easily separated by their leaf characters. *Borneocola petiolatus* has much longer petioles compared to *Borneocola argentea* (12.7–31.5 cm versus 3–6 cm). *Borneocola argenteus* also has prominently raised lateral veins on the adaxial surface of lamina, more conspicuous on dried specimens than fresh materials. This character is lacking in *Borneocola petiolatus*.

### 6. Borneocola reticosus

Taxon classificationPlantaeZingiberalesZingiberaceae

(Ridl.) Y.Y.Sam comb. nov.

urn:lsid:ipni.org:names:77158816-1

Gastrochilus reticosa Ridl., J. Straits Branch Roy. Asiat. Soc. 44: 195 (1905). Boesenbergia reticosa (Ridl.) Merr., Bibl. Enum. Born. Pl. 122 (1921). Scaphochlamys reticosa (Ridl.) R.M.Sm., Notes Roy. Bot. Gard. Edinburgh 44: 209 (1987).

#### Type.

Cultivated in Singapore Botanic Gardens, originally from Borneo, Sarawak, First Division, Bidi, 22 Nov 1904, Ridley s.n. (holotype: SING!).

#### Notes.

*Borneocola reticosus* is chosen as the type species as it is the easiest to recognise in the genus. Its reticulate lamina readily distinguishes it from other *Borneocola* species.

### 7. Borneocola salahuddinianus

Taxon classificationPlantaeZingiberalesZingiberaceae

(Meekiong, A.Ampeng & Ipor) Y.Y.Sam comb. nov.

urn:lsid:ipni.org:names:77158817-1

Scaphochlamys salahuddiniana Meekiong, A.Ampeng & Ipor, Folia Malaysiana 12(1): 22 (2011).

#### Type.

MALAYSIA. Sarawak, Kapit, Ulu Katibas, Lanjak Entimau Wildlife Sanctuary, Bukit Sepali, 30 April 2008, Meekiong MK1856 (holotype SAR; isotype Herbarium, Universiti Malaysia Sarawak.. Types not yet deposited as of 5 May 2016).

#### Note.

*Borneocola salahuddinianus* is unique amongst the Bornean species with its broadly elliptic or ovate lamina held by a long slender petiole. It is doubtful that *Borneocola salahuddinianus* is a lithophyte as observed by Meekiong et al. (2011). The plants are more of an opportunist growing on humus-rich substrate accumulated on the rocks.

### 8. Borneocola stenophyllus

Taxon classificationPlantaeZingiberalesZingiberaceae

(I.H.Ooi & S.Y.Wong) Y.Y.Sam comb. nov.

urn:lsid:ipni.org:names:77158818-1

Scaphochlamys stenophylla I.H.Ooi & S.Y.Wong, Willdenowia 44(2): 241-245 (2014).

#### Type.

MALAYSIA. Sarawak, Kuching Division, Bau, Gunung Buan, 1°33'28.9"N, 10°08'35.2"E, 92 m, 21 Nov 2013, Ooi Im Hin & Jepom ak Tisai OIH74 (holotype: SAR. Type not yet deposited as of 5 May 2016).

#### Note.

*Borneocola stenophyllus* is another new species recently discovered from Sarawak. Its grass-like leaves instantly separate it from other species in the genus.

### Incompletely known species

*Scaphochlamys anomala* (Hallier f.) R.J.Searle, Edinburgh J. Bot. 67: 85 (2010).

*Kaempferia anomala* Hallier f., Bull. Herb. Boissier 6: 357 (1898). *Gastrochilus anomalum* (Hallier f.) K.Schum. in Engler, Pflanzenr. IV, 46 (Heft 20): 92 (1904). *Boesenbergia anomala* (Hallier f.) Schltr., Repert. Spec. Nov. Regni Veg. 12: 315 (1913).

*Gastrochilus hallieri* (Hallier f.) Ridl., J. Straits Branch Roy. Asiat. Soc. 32: 109 (1899), nom. illegit.

**Type.** INDONESIA. Cultivated in Bogor, originally from Liang Gagang, Kalimantan Borneo, Hallier s.n. (original material: BO, specimen lost; lectotype (designated by Searle, 2010) Figure drawn from original Hallier’s material and published as t. IX, fig. 3, Bull. Herb. Boissier 6: 357 (1898).

**Notes.** The type, the only specimen ever collected, was lost. However, Hallier (1898) gave a very detailed description and drawing of the plant and this has convinced Searle (2010) to place it in the genus *Scaphochlamys*. The drawing, which is based on the type specimen and designated by Searle as the lectotype, is the only material that gives a glimpse of the appearance of the species. In the drawing, the flower and spirally arranged floral bracts are typical of both *Scaphochlamys* and *Borneocola*. Until another specimen is collected and is available for close examination, we prefer to retain this imperfectly known species in *Scaphochlamys*.

## Discussion

The phylogenetic analyses confirm the distinctive character of *Borneocola* and *Scaphochlamys* and their placement in the tribe Zingibereae (Figures 1, 2, 3). The *Borneocola* species form a monophyletic group which is sister to *Myxochlamys*. It is surprising to find *Borneocola* having a closer affinity to *Myxochlamys* than to *Scaphochlamys*, considering it shares more morphological similarities with *Scaphochlamys* than with *Myxochlamys*.

Morphologically, *Myxochlamys* is very different from *Borneocola*. There are two *Myxochlamys* species named so far: *Myxochlamys amphiloxa* and *Myxochlamys mullerensis* (Takano and Nagamasu 2007; Searle and Newman 2010) and a third undescribed species, also from Borneo. All three *Myxochlamys* species are very robust plants that can attain a height of 70 cm. Most *Borneocola* species examined so far are small-sized (not more than 50 cm tall), except for *Borneocola calcicola* which can grow to 60 cm tall. *Myxochlamys* has 3–10 large leaves (50–60 cm long) in each shoot whereas *Borneocola* are unifoliate and the leaves are small (less than 20 cm long except for *Borneocola calcicola*). The leaves of *Myxochlamys* are sessile compared to the conspicuously stalked leaves in *Borneocola*. The most marked difference is in the inflorescence structure. *Borneocola* has small inflorescences consisting of less than 15 fertile bracts but *Myxochlamys* has large torch-like inflorescences with easily more than 40 bracts. The bracts of *Borneocola* are membranous and marcescent, often measuring less than 2 cm long (except for *Borneocola calcicola* measuring 2.5–3.2 cm long). By contrast, the floral bracts of *Myxochlamys* are coriaceous, persistent, measuring 2.5–5 cm long and most notably are covered with transparent slimy mucilage. In addition, the unique versatile anthers of *Myxochlamys*, a rare feature in the Zingiberaceae, are distinct from the adnate anthers in *Borneocola* and also from all its sister genera. Based on morphological features, *Myxochlamys* is more similar to *Scaphochlamys*, the closest being *Scaphochlamys grandis*. Both have large sessile leaves and decurrent lamina base, large, coriaceous and persistent floral bracts,their bracts being concave with reflexed and spreading apices.

Based on morphology, *Borneocola* is also similar to *Distichochlamys*. However, *Distichochlamys* is distinguished from *Borneocola*, *Myxochlamys* and *Scaphochlamys* by its unique tubular bracteoles, floral tube without a groove on the inner surface and trilocular ovary (Newman 1995). Other characteristics such as distichous floral bracts, 2-keeled bracteoles, thecae without basal spurs have been observed in the three closely allied sister genera in this study (Table 2).

**Table 2.** Comparison between the morphological characters of *Borneocola*, *Distichochlamys*, *Myxochlamys* and *Scaphochlamys*.

Morphology	*Borneocola*	*Distichochlamys*	*Myxochlamys*	*Scaphochlamys*
Plant height	to 50(–60) cm	to 60 cm	70 cm	to 100 cm
Number of leaf in each leafy shoot	1	1–3	3–10	1–7
Bladeless sheath	Papery, drying fast	Papery, decaying fast	Not mentioned	Coriaceous, persistent
Leaf (cm)	6–37 × 1–18; petiolate	15–28 × 8.3–14.5; petiolate	50–65 × 7–17; sessile	9–50 × 3–24; petiolate or sessile
Inflorescence height (cm)	3–11.5	to 15.5	6.5–18	4–28
Number of floral bracts	3–13	7–13	c. 40	4–44
Arrangement of floral bracts	Spiral, rarely distichous	Distichous	Spiral	Spiral, rarely distichous
Floral bracts	Thin, translucent, without mucilage; drying fast	Without mucilage; persistent	Coriaceous, with mucilage; persistent	Coriaceous, without mucilage; persistent
Flowers	In cincinni	In cincinni	Solitary	In cincinni
First bracteole	Open to base, 2-keeled	Tubular, 2-keeled	Open to base, 2-keeled	Open to base, 2-keeled
Floral tube	With a groove in inner surface, glabrous to puberulent externally	Without a groove in inner surface, glabrous externally	With a groove in inner surface, glabrous externally	With a groove in inner surface, glabrous externally
Labellum	Bilobed, rarely entire, not concave; without coloured streaks beside median band	Bilobed, not concave; without coloured streaks beside median band	Not bilobed, entire, concave; without coloured streaks beside median band	Bilobed, rarely entire, not concave; with coloured streaks beside median band
Thecae	Spurs absent or with short free basal spurs	Spurs absent	Spurs present and long	Spurs absent or with short free basal spurs
Anther	Adnate	Adnate	Versatile	Adnate
Ovary	Unilocular with basal placentation	Trilocular with axile placentation	Unilocular with basal placentation	Unilocular with basal placentation
Chromosome number	2*n*=10 (Šída et al., unpublished data)	2*n*=26	—	2*n*=28
Geographical distribution	Borneo	Vietnam	Borneo	Southern Thailand, Peninsular Malaysia, Sumatra, Borneo

*Haplochorema* K.Schum. is another small-sized genus endemic to Borneo, which can be mistaken for *Borneocola*. It has short and few-flowered inflorescences as in *Borneocola* but its flowers appear somewhat quadrate with the labellum and lateral staminodes held flat, more resembling *Kaempferia* L. *Haplochorema* has distichous floral bracts, single-flowered cincinni and the flowering proceeds from apex to base, to name some of the characters which distinguish it from *Borneocola*. In fact, the genus is more allied to *Boesenbergia* Kuntze than *Borneocola*.

*Borneocola* is morphologically most similar to *Scaphochlamys* but both can be distinguished by the texture of the bladeless sheath and floral bracts, variegation on the labellum, indumentum on the floral tube and the stigma shape. The current study recognises eight *Borneocola* species while *Scaphochlamys gracilipes*, *Scaphochlamys polyphylla* B.L.Burtt & R.Sm., *Scaphochlamys limiana* Meekiong & K.Yazid and *Scaphochlamys samunsamensis* Meekiong & Hidir from Borneo remain in the genus *Scaphochlamys*. There are no recent collections of *Scaphochlamys gracilipes* but the lax inflorescence and persistent floral bracts in the type specimens clearly distinguish it from the *Borneocola* species. *Scaphochlamys polyphylla*, *Scaphochlamys limiana* and *Scaphochlamys samunsamensis* can be readily distinguished from the *Borneocola* species by their papery bladeless sheath and large, green or green tinged red, coriaceous floral bracts. This shows that the distinct morphologies that separate *Borneocola* and *Scaphochlamys* are significant and are also supported by the phylogenetic analyses (Figures 1, 2 and 3). An anatomical study on the leaves also discovered some characteristics that separate *Borneocola* from *Scaphochlamys* (Norhati, pers. comm).

The morphology of *Borneocola* is very similar to *Scaphochlamys* but, combining both, necessitates synonymising *Myxochlamys* and possibly *Distichochlamys* and this will result in a very heterogenous genus. A similar situation is observed in the naming of *Newmania* N.S. Lý & Škorničk, a genus very similar in morphology to *Haniffia* Holttum but appears as its sister group in the molecular phylogenetic analyses. The authors decided against placing *Newmania* under *Haniffia* which would create a heterogenous group. The current description of *Borneocola* is further supported by the chromosome number with 2*n*=10 (Šída et al., unpublished data), different from *Distichochlamys* (2*n*=26) and *Scaphochlamys* (2*n*=28). Such significant differences in molecular data and chromosome number have conclusively supported the circumscription of the new genus *Borneocola*.

### Key to the genera of the Zingibereae tribe in Borneo

**Table:** 

1	Inflorescence arising directly from the rhizome on a leafless shoot	**2**
–	Inflorescence emerging at the terminal of the leafy shoot	**3**
2	Distinct swelling at the base of the petiole; anther with long extended crest wrapped around the style	***Zingiber***
–	No swelling at the base of the petiole; anther crest short, not long extended and not wrapped around the style	***Haniffia***
3	Flowers with versatile anther	**4**
–	Flowers with adnate anther	**5**
4	Inflorescence with few to many floral bracts, bracts mucilage	***Myxochlamys***
–	Inflorescence with one single large floral bract, bracts not mucilage	***Camptandra***
5	Flowers opening from top to bottom of inflorescence	**6**
–	Flowers opening from bottom to top of inflorescence	**7**
6	Flowers appearing quadrate with the two petaloid staminodes	***Haplochorema***
–	Flowers no quadrate appearance, staminodes not petaloid	***Boesenbergia***
7	Flowers with long narrow corolla lobes and long exserted stamens	***Hedychium***
–	Flowers without such features	**8**
8	Floral bracts coriaceous and persistent, labellum with coloured streaks on both sides of the median band	***Scaphochlamys***
–	Floral bracts thin, translucent and marcescent, labellum without coloured streaks on both sides of the median band	***Borneocola***

## Supplementary Material

XML Treatment for Borneocola

XML Treatment for Borneocola argenteus

XML Treatment for Borneocola biru

XML Treatment for Borneocola calcicola

XML Treatment for Borneocola iporii

XML Treatment for Borneocola petiolatus

XML Treatment for Borneocola reticosus

XML Treatment for Borneocola salahuddinianus

XML Treatment for Borneocola stenophyllus
